# PERCEIVED AND OBJECTIVE EXECUTIVE DYSFUNCTION JOINTLY PREDICT BRAKING REACTION TIME IN OLDER DRIVERS

**DOI:** 10.1093/geroni/igac059.653

**Published:** 2022-12-20

**Authors:** Caitlin Pope, Tyler Bell, Benjamin McManus, Despina Stavrinos

**Affiliations:** University of Kentucky, Lexington, Kentucky, United States; University of California, San Diego, La Jolla, California, United States; University of Alabama at Birmingham, Birmingham, Alabama, United States; University of Alabama at Birmingham, Birmingham, Alabama, United States

## Abstract

Motor vehicle crashes are a leading cause of injury and death for older adults in the United States. Braking reaction time (BRT), how quickly a driver responds to the situational demands of driving, is a known predictor of driver fitness and hypothesized as sensitive to difficulties with executive functioning. Unclear is how BRT may vary across different levels of executive functioning, including objective test performance and perceived executive dysfunction, and if awareness of executive dysfunction in the presence of objective difficulties jointly predicts BRT. Using data from a simulated driving study, 50 adults aged 65-94 years old (49.1% female) completed computerized EF tests (inhibition [Stroop], working memory [Automated Operation Span]), the Behavior Rating Inventory of Executive Function, the Big Five Inventory, and a simulated drive. Multilevel modeling analyses controlling for covariates of empirical and design relevance (simulated road segment, age, gender, and neuroticism) were performed. Our results show a significant interaction between perceived executive dysfunction and objective test performance on BRT. Specifically, associations with BRT were stronger for individuals who perceived more executive dysfunction in the presence of worse inhibition (b = 0.79, 95%CI = 0.27, 1.30) and worse working memory (b = 0.94, 95%CI = 0.26, 1.62). Findings provide further justification for the role of executive functioning in monitoring driver fitness in older age. Future directions and implications are discussed.

